# Trajectories of child mental health, physical activity and screen-time during the COVID-19 pandemic considering different family situations: results from a longitudinal birth cohort

**DOI:** 10.1186/s13034-023-00581-3

**Published:** 2023-03-10

**Authors:** Deborah Kurz, Stefanie Braig, Jon Genuneit, Dietrich Rothenbacher

**Affiliations:** 1 Institute of Epidemiology and Medical Biometry, Ulm University, Helmholtzstrasse 22, 89081 Ulm, Germany; 2 Pediatric Epidemiology, Clinic and Polyclinic for Pediatric and Adolescent Medicine, Department of Pediatrics, Medical Faculty, Leipzig University, Liebigstraße 20a, 04103 Leipzig, Germany

**Keywords:** COVID-19, Child health, Mental health, Quality of life, Wellbeing, SDQ, KINDL, Screen-time

## Abstract

**Background:**

Many authors have described a significant mental health burden on children and adolescents during the COVID-19 pandemic, possibly moderated by social disparities. This analysis explores whether pre-pandemic family circumstances might be related to different aspects of child health during the pandemic.

**Methods:**

We analyzed trajectories of health-related outcomes in children aged 5 to 9 years (T7 to T11) using the Ulm SPATZ Health study, a population based birth cohort study (baseline 04/2012–05/2013) conducted in the South of Germany. Outcomes were children’s mental health, quality of life, and lifestyle, such as screen time and physical activity. We conducted descriptive statistics of maternal and child characteristics before and throughout the pandemic. We defined three different groups of pre-pandemic family situations and used adjusted mixed models to estimate differences in means associated with the time during the pandemic vs. before the pandemic in (a) all children and in (b) children belonging to specific pre-pandemic family situations.

**Results:**

We analyzed data from n = 588 children from whom at least one questionnaire was completed between T7 and T11. When not considering the pre-pandemic family situation, adjusted mixed models showed statistically significant lower mean scores of health-related quality of life among girls during vs. before the COVID-19 pandemic (difference in means (b): − 3.9 (95% confidence interval (CI): − 6.4, − 1.4). There were no substantial differences in mental health, screen time, or physical activity in boys or girls. When considering pre-pandemic family situations, boys with mothers having symptoms of depression or anxiety showed a substantial loss of health-related quality of life on the subscale of friends (b: − 10.5 (95% CI: − 19.7, − 1.4)). Among girls in this group, 60% of the 15 assessed outcomes were negatively associated with a remarkable loss in health-related quality of life (e.g., KINDL-*physical well-being* difference in means: − 12.2 (95% CI: − 18.9, − 5.4)). Furthermore, a substantial increase in screen time was found (+ 2.9 h (95% CI: 0.3, 5.6)).

**Conclusion:**

Our results suggest that the health (and behavior) of primary school-aged children is possibly impacted by the COVID-19 pandemic, with adverse consequences differing by gender and very likely by the pre-pandemic family situation. Especially in girls having a mother with depression or anxiety symptoms, the adverse consequences of the pandemic on mental health seem to be aggregated. Boys showed fewer adverse trajectories, and it needs to be further assessed which factors exactly are behind the (socio-economic) factors, such as maternal working habits and limited living space, when analyzing the effect of the pandemic on children’s health.

**Supplementary Information:**

The online version contains supplementary material available at 10.1186/s13034-023-00581-3.

## Introduction

Responding to the COVID-19 pandemic has been very challenging for children and parents. The costs (e.g., in terms of consequences on physical and mental health) in different parts of society are still poorly understood or yet insufficiently quantified. Therefore, careful assessment of lifestyle and psychological changes related to the pandemic is needed [1–4], as is a better understanding of potential moderators and mediators of the potential associations [3, 5]. This is very likely necessary to identify the children’s specific needs during a pandemic, as well as during the long phase of recovery from the pandemic situation back to normal [6–8]. Such insights can guide policy makers in quick and fit-for-purpose health promotion strategies that target different children’s needs and vulnerable groups.

Many authors have described a significant mental health burden of children and adolescents living in Germany during the COVID-19 pandemic [2, 9–11]. Two-thirds of the children included in a study conducted between May 26 and June 10, 2020, reported to be highly burdened by the pandemic and to have more mental health problems and higher anxiety levels than before the pandemic [2]. Additionally, data from the Netherlands showed decreased mental health in children and adolescents [12]. Similar results were found for adolescents living in Australia [13], primary school-aged children living in Wales [11], and students in China [14, 15]. These results are underpinned by a meta-analysis and a systematic review suggesting that during the COVID-19 pandemic, children’s mental health was generally negatively impacted [16, 17]. Even if there are also some health-related outcomes that might be affected positively by the COVID-19 pandemic, such as higher levels of resilience or experiences of positive feelings such as empathy, gratefulness, connection with others, kindness, or calmness [18], the most outcomes assessed were negatively impacted [14, 17, 19], e.g., sleep [20, 21], screen-time [9, 20, 22], and physical activity. [9, 22–24]

However, the pandemic may have not affected all families and children in the same way. Social disparities, such as a low socio-economic status (in terms of education of the head/s of the household [11, 25, 26], limited living space [2, 27], or unstable jobs [7]), may be related to greater adverse impacts on children’s health. Additionally, maternal health conditions, such as mental health and other chronic diseases, might lead (a) to different trajectories of child health and (b) those trajectories might be affected differently by the pandemic. For example, it was shown that maternal and paternal mental health during the pandemic was related to the child mental health indexed with emotional and behavioral difficulties score assessed with the German version of the Strengths and Difficulties Questionnaire (SDQ) in their children [11], suggesting complex and reciprocal associations.

Since there are few data on the impact of a child’s living or family situation and the potentially harmful effects of the pandemic responses on a child’s health and well-being [28], the main goal of this analysis was to explore whether pre-pandemic family circumstances (housing density, maternal health conditions, and maternal working habits) might be related to different aspects of child health during the pandemic.

The theoretical basis of this work is guided by the framework from Prime, Wade, and Brown developed in 2020 describing risk and resilience of family well-being during the COVID-19 pandemic taking pre-pandemic family situations into account [29]. The framework explains how a disruption of the family system caused by the pandemic relates to child mental health.

Concerning housing density, our work is guided by the housing-health relationship suggesting that the space available per individual [30] and housing quality can affect mental health [30, 31] which might be especially true for children during the pandemic [32]. Several studies have explored how housing environment and mental health was associated during the COVID-19 pandemic [33–36]. Most of them found that limited living space was associated with poorer mental health outcomes [34–36]. However, Keller et al. found that high housing density was linked to poorer mental health outcomes in adults (> 25 years) but higher mental health in younger individuals (< 25 years). [33]

We identified three different groups of pre-pandemic family situations and analyzed child health trajectories before and throughout the pandemic in these groups. Outcomes were children’s mental health, quality of life, and lifestyle, e.g., screen time and physical activity in a longitudinal manner. We used data from a longitudinal birth cohort study, the Ulm SPATZ study, and analyzed children aged 5 to 9 years. To minimize reverse causality, pre-pandemic data were used for the definition of specific family situations.

## Methods

### Study design, study population and ethical approval

SPATZ is a population-based longitudinal (birth-) cohort study conducted in Ulm in southern Germany that started recruiting newborns and their mothers during hospitalization after delivery in the Department of Gynecology and Obstetrics, University Medical Center Ulm, in 2012 (which was the only maternity hospital in Ulm at this time). The baseline took place from April 2012 to May 2013 (wave T0) (overall response was 49%). Details are described elsewhere [37]. Ethical approval was obtained from the Ethics Board of Ulm University (no. 311/11).

### Sampling procedure and sample description

**Fig. 1:**
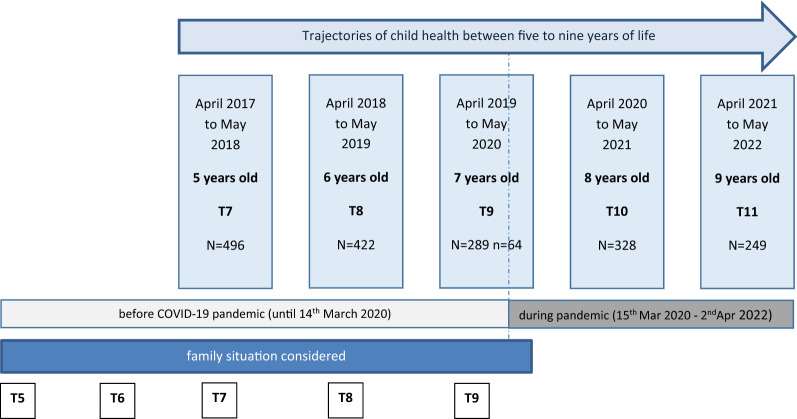
Study design of trajectories of child health in light of the COVID-19 pandemic (T7-T11) taking family situation into account

Children aged 5–9 years who completed at least one questionnaire in the SPATZ study were included in this analysis. COVID-19 pandemic started in the first or second year of school (children aged 6 or 7 years). Data for the pre-pandemic family situation were taken from the follow-up waves T5-T9. See Fig. 1 for details of the study design and data considered.

### COVID-19 pandemic

As the starting point of the COVID-19 pandemic in Germany, we chose March 15, 2020. Notably, schools closed on March 16, 2020, and the first economic shutdown was on March 22, 2020. We assumed an ongoing pandemic throughout April 2, 2022, as throughout the whole time public health measures were established to mitigate the spread of the COVID-19 pandemic. Those measures ranged from mask mandates (also in schools and public), home office regulations for parents, limited leisure time activities, travel restrictions, partial home-schooling, limited possibilities for social contacts (also in schools) and unusual structure of school-days (e.g., ventilation, split schoolyards to prevent transmission of the virus, daily fast-testing routines). In this analysis, April 2, 2022 marks the “last day of the pandemic” (i.e. last longitudinal data point considered) since almost all public health measures to mitigate the spread of the COVID-19 pandemic were discontinued in the study region Baden-Württemberg at that time [38]. The pre-pandemic family situation was taken into account between T5 and T9 to minimize reverse causality. In particular, since it is possible that parental outcomes during the pandemic are influenced by changing offspring outcomes during the pandemic, the results of this approach can be clearly interpreted based on family situation before the pandemic.

### Measures and outcome variables

The main outcome variables of interest were parental reports of (1) children’s health-related quality of life (German version of the KINDL-R questionnaire [39, 40]). This questionnaire consists of 24 items covering six dimensions: family, physical well-being, emotional well-being, self-esteem, friends, and school. For the total score (outcome “health-related quality of life”), all 24 items are summed up and transformed to a 0–100 scale; higher values represent better quality of life.

Another outcome variable assessed was (2) child mental health indexed with emotional and behavioral difficulties score assessed with the German version of the Strengths and Difficulties Questionnaire (SDQ) [41]. This questionnaire consists of 25 items covering 5 subscales (emotion, behavior, hyperactive, peers and prosocial behavior). For the total difficulties score, all subscales except prosocial behavior are summed up (20 items); a higher score indicates more difficulties.

A further outcome variable was (3) screen time which was assessed via self-administered parental questionnaires. For screen time, the items covered time spent with TV/DVD (also via computer/smartphone), time spent with computer games/game consoles (also via smartphone), and time spent with other uses of internet/computer (also via smartphone). Data were average hours on school days and on weekends.

The last outcome variable included was (4) physical activity assessed with a short self-administered parental questionnaire from Bayer [42]. The questionnaire consists of seven items to calculate the child’s physical-activity score. The number of items answered as physically inactive was subtracted from the number of items answered as physically active. Consequently, the score can range from − 7 to + 7, with a higher score representing more physical activity.

All questionnaires used were validated, except for screen time, although the categories were similar to those used in another large German population-based study (KIGGS) [43].

### Maternal data for assessing pre-pandemic family situation

We used maternal questionnaire data of the FU waves T5-T9 to define three different groups of pre-pandemic family situations to allow simple identification of children possibly at risk. A child belonged to a specific group if maternal criteria for the group were fulfilled. Table 1 shows how groups of different pre-pandemic family situations were defined.

**Table 1:** Groups of considered pre-pandemic family situations based on maternal questionnaire data of follow-up waves T5-T9

Group #	Group name	Definition	Comment	N girls	N boys
	Overall			300	288
#1	Depression or anxiety symptoms of mother	Mother has at least two times HADS_D score ≥ 8 or two times HADS_A score ≥ 8 in FU-waves T5-T8	Group of children in which mother has either symptoms of depression or anxiety	64	67
#2	Housing density	High housing density for at least two years between T5-T9	Living space per person (m^2^/person) < median of specific FU-wave: T5 < 32.5, T6 < 32.5, T7 < 32.0, T8 < 32.5, T9 < 33.5	148	131
#3	Working days of mother	Mother works ≥ 5 days per week for at least one year in FU-waves T6-T9	–	150	115

*FU* follow-up

Maternal pre-pandemic mental health was indexed by the Hospital Anxiety and Depression (HADS)-scale. The HADS questionnaire [44, 45] is a 14-item screening measure with two subscales assessing symptoms of anxiety and depression. Scores on each subscale range from 0 to 21. A score between 8 and 10 indicates moderate levels of symptoms, and a score between 11 and 21 indicates severe levels of symptoms [46]. The questionnaire is also validated in the German language and can be used in the general population [46].

#### Statistical analysis

We conducted descriptive statistics and estimated the trajectories of outcomes between T7 and T11 (i.e. child age 5–9 years). The maximum number of measurement points used per individual was five, and the minimum number of measurement points per individual to be included in the analysis was one.

We used mixed models, assuming an unstructured covariance matrix, to estimate differences in means associated with the time during the pandemic vs. before the pandemic. Adjustment variables were child age, maternal educational attainment (duration of school education < 12 years/duration of school education ≥ 12 years) and maternal nationality (German/Non-German). The intercept represents the individual score at baseline. The analysis was stratified by gender and was performed using SAS® 9.4 (The SAS Institute, Cary, NC, USA).

## Results

### Descriptive results

Maternal pre-pandemic data defining the family situation are described in Table 2. The majority of mothers had German nationality (88.4%), and more than half of them had ≥ 12 years of school education. Approximately 30% of mothers worked 5 days per week (at all time points T5-T11). The mean housing density (m^2^/person) was 35.1 (SD 13.0) at T5 and stayed more or less the same until T9. Further descriptive data are reported in Additional file 1:Table S1**.** A larger share of mothers had noticeable (i.e. ≥ 8) anxiety scores (approximately 20–30%) than noticeable depression scores (approximately 10–15%).

**Table 2:** Maternal data considered for pre-pandemic family situation

Wave	**T0**	**T5**	**T6**	**T7**	**T8**	**T9**
Duration of data sampling for specific follow-up (FU) wave	04/12—05/13	04/15–07/16	04/16—07/17	04/17—07/18	04/18—07/19	04/19—07/20
Mothers, N	970	625	546	477	408	341
Age (n observations)	(961)	(625)	(546)	(477)	(408)	(341)
Mean (SD)	32.7 (4.8)	36.3 (4.4)	37.4 (4.5)	38.4 (4.4)	40.6 (4.4)	40.6 (4.4)
Nationality	(970)					
German	857 (88.4)					
Non-German	105 (10.8)					
Missing	8 (0.8)					
School education years	(970)					
≤ 9 years education	93 (9.6)					
10 to 11 years education	297 (30.6)					
≥ 12 years education	561 (57.9)					
Missing	19 (2.0)					
Number of regular weekly working days, N (%)	–	–	(546)	(477)	(408)	(345)
< 5 days/week	–	–	364 (66.7)	309 (64.2)	253 (62.0)	208 (60.3)
≥ 5 days/week	–	–	167 (30.6)	154 (32.2)	143 (35.1)	128 (37.1)
Missing	–	–	15 (2.8)	19 (3.7)	12 (3.0)	9 (2.6)
Housing density, m^2^/person	–	(616)	(531)	(465)	(402)	(328)
Mean (SD), Median (Q1, Q3)	–	35.1 (13.0), 32.5 (26.7, 40.0)	34.9 (12.9), 32.5 (26.7, 40.0)	34.9 (12.1), 32.0 (27.5, 40.0)	34.7 (11.3), 32.5 (27.5, 40.0)	35.8 (11.4), 33.5 (28.0, 40.0)
Hospital Anxiety and Depression Scale (HADS)	(957)	(622)	(545)	(475)	(406)	–
Mean (SD), Median (Q1, Q3)	7.3 (5.0), 6.0 (4.0, 10.0)	8.6 (5.4), 8.0 (5.0, 12.0)	8.9 (5.6), 8.0 (5.0, 12.0)	8.7 (5.6), 8.0 (5.0, 12.0)	9.4 (5.7), 9.0 (5.0, 13.0)	–
HADS_A ≥ 8, N (%)	176 (18.4)	141 (22.6)	139 (25.5)	116 (24.4)	114 (28.1)	–
HADS_D ≥ 8, N (%)	46 (4.8)	60 (9.6)	50 (9.2)	41 (8.6)	42 (10.3)	–

Table 3 shows descriptive data for the analyzed children (n = 588 children for whom at least one questionnaire was completed during T7 and T11). At T7, children were on average 5.1 years old. The mother was the main care person for the child in almost all families (T7: 92.7%, T11: 93.0%). The health-related quality of life of children was 80.6 (SD 0.4) at T7, more or less the same for the consecutive year (T8), followed by a rise before the pandemic (84.2 (SD 0.5)) and a drop with the start of the pandemic (81.5 (SD 1.1)). This pattern is reflected in all six subscales of health-related quality of life (Table 3, Additional file 1:Table S2). The total difficulties score (SDQ) also changed with the age of the child (i.e. FU-waves T7-T11) and showed an improved difficulties score, though the descriptive trend discontinued when the pandemic started: the T9-pre-pandemic total difficulties score was 6.4 (SD 0.3), whereas the T9-during-pandemic total difficulties score was 7.5 (SD 0.7). This score remained high at T10 (7.3 (SD 0.3)) and slightly dropped at T11 (6.7 (SD 0.3)). The five subscales of the SDQ showed more or less the same pattern as the overall total difficulties score. The physical activity of children was slightly higher immediately after the start of the pandemic (1.8 (SD 0.4)) than that of same-aged children before the pandemic (1.6 (SD 0.2)) (Table 3). Screen time also increased when the pandemic started (6.9 (SD 0.3) h/week to 8.8 (SD 0.9) h/week). Screen time further increased with the ongoing pandemic and the age of the child to 9.3 (SD 0.4) h/week and 10.9 (SD 0.5) h/week in 10- and 11-year-old children, respectively.

**Table 3:** Children data for waves 7, 9, and 11 (data for waves 8, and 10 shown in Additional file 1: Table S2)

Wave (Age child)	T7 (5 years old)	T9 (7 years old)		T11 (9 years old)
Duration of data sampling for specific follow-up (FU) wave	04/17—07/18	04/19—07/20		04/21—07/22
Children, N	496	353		249
Set of twins	18	11		5
Age (n observations)	(496)	(353)		(249)
Mean (SD)	5.1 (0.1)	7.1 (0.1)		9.1 (0.1)
SARS-COV-2-infection	–	–		(249)
Ever, N (%)	–	–		15 (6.0)
Health related quality of life (KINDL)^a^	*pre-pandemic* (479)	*pre-pandemic* (280)	*during pandemic* (57)	*during pandemic* (242)
Total score, Mean (SD), Median (Q1, Q3)	80.6 (0.4), 81.3 (76.0, 86.5)	84.2 (0.5), 84.4 (79.2, 90.1)	81.5 (1.1), 81.3 (77.1, 86.5)	81.9 (0.6), 83.3 (77.1, 88.5)
N	(494)	(289)	(62)	(245)
Family, Mean (SD), Median (Q1, Q3)	82.5 (0.5), 81.3 (75.0, 93.8)	83.1 (0.7), 81.3 (75.0, 93.8)	79.3 (1.5), 81.3 (75, 87.5)	81.8 (0.8), 81.3 (75.0, 93.8)
N	(494)	(289)	(62)	(245)
Physical well-being, Mean (SD), Median (Q1, Q3)	84.3 (0.6), 87.5 (75.0, 93.8)	86.7 (0.7), 87.5 (81.3, 93.8)	87.3 (1.2), 87.5 (81.3, 93.8)	87.3 (0.9), 93.8 (81.3, 100.0)
N	(494)	(289)	(62)	(245)
Emotional well-being, Mean (SD), Median (Q1, Q3)	86.0 (0.5), 87.5 (81.3, 93.8)	85.6 (0.7), 87.5 (75, 93.8)	83.7 (1.4), 84.4 (75, 87.5)	82.6 (0.8), 87.5 (75.0, 93.8)
N	(492)	(289)	(62)	(245)
Self-esteem, Mean (SD), Median (Q1, Q3)	79.4 (0.6), 78.1 (75.0, 87.5)	78.9 (0.8), 75.0 (68.8, 87.5)	74.8 (1.9), 75.0 (68.8, 81.3)	76.8 (0.9), 75.0 (68.8, 87.5)
N	(493)	(289)	(58)	(244)
Friends, Mean (SD), Median (Q1, Q3)	81.8 (0.5), 81.3 (75.0, 87.5)	80.7 (0.7), 81.3 (75.0, 87.5)	75.4 (2.5), 75 (68.8, 87.5)	80.9 (0.9), 81.3 (75.0, 93.8)
N	(482)	(280)	(58)	(243)
School, Mean (SD), Median (Q1, Q3)	69.7 (0.4), 68.8 (62.5, 75.0)	89.5 (0.6), 93.8 (87.5, 100.0)	87.4 (1.5), 87.5 (81.3, 93.8)	81.8 (0.9), 81.3 (75.0, 93.8)
SDQ Strengths and Difficulties Questionnaire^b^	(493)	(289)	(64)	(248)
Total Difficulties Score, Mean (SD), Median (Q1, Q3)	7.1 (0.2), 6.3 (4.0, 9.0)	6.4 (0.3), 6.0 (3.0, 9.0)	7.5 (0.7), 6.0 (4.0, 10.0)	6.7 (0.3), 6.0 (3.0, 10.0)
SDQ emotion, Mean (SD)	1.4 (0.07)	1.3 (0.09)	1.1 (0.2)	1.5 (0.1)
SDQ behavior, Mean (SD)	1.9 (0.07)	1.5 (0.09)	2.0 (0.2)	1.5 (0.1)
SDQ hyperactive, Mean (SD)	2.7 (0.1)	2.6 (0.1)	3.3 (0.3)	2.8 (0.2)
SDQ peers, Mean (SD)	1.1 (0.07)	1.0 (0.09)	1.1 (0.2)	1.0 (0.09)
SDQ prosocial, Mean (SD)	7.9 (0.08)	8.1 (0.1)	7.7 (0.3)	8.2 (0.1)
Physical Activity Score^c^	(495)	(289)	(64)	(249)
Mean (SD), Median (Q1, Q3)	0.4 (0.1), 1.0 (-1.0, 3.0)	1.6 (0.2), 1.0 (-1.0, 3.0)	1.8 (0.4), 2.0 (-1.0, 3.0)	1.2 (0.2), 1.0 (-1.0, 3.0)
Screen-time (h/week)^d^	(491)	(287)	(62)	(248)
Mean (SD), Median (Q1, Q3)	5.4 (0.2), 4.5 (2.0, 8.0)	6.9 (0.3), 5.0 (3.0, 9.0)	8.8 (0.9), 7.0 (4.1, 11.5)	10.9 (0.5), 8.6 (5.5, 14.5)
Time spent with books (h/week)^e^	(491)	(287)	(62)	(248)
Mean (SD), Median (Q1, Q3)	6.7 (0.2), 6.0 (3.5, 8.4)	6.7 (0.3), 5.3 (3.5, 8.4)	6.2 (0.6), 4.5 (3.5, 8.0)	6.7 (0.3), 5.4 (3.5, 9.0)

h = hours; SD = standard deviation. Q1 = First Quartil; Q3 = Third Quartil

^a^KINDL questionnaire: higher values indicate more health-related quality of life

^b^Strengths and Difficulties Questionnaire (SDQ): higher values indicate more emotional and behavioral difficulties, except for SDQ-prosocial-score, where it is the inverse

^c^Physical activity-score: Higher score more physical active. The score indicates how many items answered with "physically active" outweigh the items answered with "physically inactive". Score from -7 to + 7

^d^Including time spent with TV/DVD (also via computer/smartphone), time spent with computer games/game consoles (also via smartphone), time spent with other use of internet/computer (also via smartphone)

^e^Either read by themselves or read to them by someone else

### Analytical results

#### Overall results in girls and boys

We analyzed data from n = 588 children for whom at least one questionnaire was completed during T7 and T11 (median number of measurement points used per child 4.0 (Q1 2.0, Q3 4.0), n = 102 children were included with one measurement point). Adjusted mixed models showed statistically significant lower mean score (difference in means) of health-related quality of life among girls during vs. before the COVID-19 pandemic: KINDL total score − 3.9 (95% confidence interval (CI): − 6.4, − 1.4) (Table 4). Similar results were found among girls for the KINDL subscales *family*: − 5.0 (95% CI: − 8.2, − 1.7), *friends*: − 7.6 (− 11.8, 3.5), and *school*: − 4.9 (95% CI: − 8.9, − 0.9). There were no substantial differences in mental health indexed with the SDQ, either in boys or in girls. Furthermore, no differences were found in weekly screen time or physical activity when comparing the time during vs. before the pandemic.

**Table 4:** Results of adjusted mixed models: differences in means comparing pre- and during pandemic outcomes of child health in all children aged 5–9 years in SPATZ (FU-waves T7-T11) n = 300 girls (877 observations: 308 during pandemic), n = 288 boys (846 observations: 300 during pandemic) (overall model)

Outcomes of child health	Boys		Girls	
	Difference in means (95% CI)	p-value	Difference in means (95% CI)	p-value
KINDL total score	− 1.1 (− 3.7, 1.4)	0.4	− **3.9 (**− **6.4, **− **1.4)**	**0.002**
KINDL family	− 1.5 (− 5.1, 2.1)	0.4	− **5.0 (**− **8.2, **− **1.7)**	**0.007**
KINDL physical well-being	2.5 (− 1.5, 6.5)	0.3	− 0.6 (− 4.9, 3.7)	0.8
KINDL emotional well-being	− 1.6 (− 5.7, 2.5)	0.4	− 3.2 (− 6.4, 0.02)	0.05
KINDL self-esteem	− 2.8 (− 6.6, 1.0)	0.1	− 3.9 (− 8.0, 0.3)	0.07
KINDL friends	− 4.1 (− 8.7, 0.5)	0.08	− **7.6 (**− **11.8, **− **3.5)**	** < 0.001**
KINDL school	1.0 (− 3.0, 4.9)	0.6	− **4.9 (**− **8.9, **− **0.9)**	**0.02**
SDQ total score	0.8 (− 0.4, 2.0)	0.2	0.4 (− 0.7, 1.6)	0.5
SDQ emotion	− 0.3 (− 0.7, 0.1)	0.1	− 0.02 (− 0.4, 0.4)	0.9
SDQ behavior	0.3 (− 0.1, 0.8)	0.1	0.3 (− 0.1, 0.7)	0.1
SDQ hyperactive	0.4 (− 0.2, 1.0)	0.2	0.3 (− 0.3, 0.8)	0.3
SDQ peers	0.4 (− 0.1, 0.8)	0.1	− 0.09 (− 0.5, 0.3)	0.7
SDQ prosocial	0.1 (− 0.4, 0.6)	0.7	− 0.3 (− 0.8, 0.2)	0.2
Screen-time (h/week)	1.2 (− 0.8, 3.3)	0.2	− 0.06 (− 1.2, 1.2)	0.9
Physical activity-score	0.4 (− 0.5, 1.3)	0.4	0.5 (− 0.3, 1.4)	0.2

P-values <0.05 are in bold and signify a statistically significant difference

Models were adjusted for age of child, maternal educational attainment and maternal nationality

KINDL-Scores: Higher values indicate more quality of life

SDQ-Scores: Higher values indicate more difficulties, except for SDQ-prosocial-score, where it is the inverse. Physical activity-score: Higher scores indicatemore physical activity

#### Considering different pre-pandemic family situations: Groups 1–3

Group 1 (Table 5): In boys, a substantial loss of health-related quality of life in the subscale *friends* was found: − 10.5 (95% CI: − 19.7, − 1.4). However, this was the only outcome among boys in this group that was associated with the pandemic. In contrast, for girls, nine of the 15 outcomes assessed were negatively associated with the pandemic: e.g., KINDL subscale *physical well-being* dropped by − 12.2 (95% CI: − 18.9, − 5.4), followed by *family* − 11.5 (95% CI: − 18.2, − 4.8). Girls also showed a substantial increase in screen time: + 2.9 h (95% CI: 0.3, 5.6).

**Table 5:** Results of adjusted mixed models in Group 1: Differences in means comparing pre- and during pandemic outcomes of child health in children with mothers experiencing symptoms of depression or anxiety, n = 64 girls (n = 219 observations: n = 77 during pandemic), n = 67 boys (n = 214 observations: 74 during pandemic)

Outcomes of child health	Boys		Girls	
	Difference in means (95% CI)	p-value	Difference in means (95% CI)	p-value
KINDL total score	− 3.3 (− 8.2, 1.7)	0.2	− **7.8 (**− **12.6, **− **3.0)**	**0.002**
KINDL family	− 2.9 (− 10.9, 5.1)	0.5	− **11.5 (**− **18.2, **− **4.8)**	**0.001**
KINDL physical well-being	3.3 (− 3.7, 10.4)	0.4	− **12.2 (**− **18.9, **− **5.4)**	** < 0.001**
KINDL emotional well-being	− 3.6 (− 11.9, 4.7)	0.4	− **8.4 (**− **15.7, **− **1.0)**	**0.03**
KINDL self-esteem	− 0.5 (− 8.1, 7.0)	0.9	− 5.2 (− 14.4, 3.9)	0.3
KINDL friends	− **10.5 (**− **19.7, **− **1.4)**	**0.02**	− **10.3 (**− **19.2, **− **1.4)**	**0.02**
KINDL school	− 1.4 (− 10.0, 7.3)	0.8	− **7.3 (**− **13.8, **− **0.8)**	**0.03**
SDQ total score	− 1.8 (− 4.1, 0.5)	0.1	1.5 (− 0.9, 3.9)	0.2
SDQ emotion	− 0.9 (− 1.9, 0.1)	0.09	0.5 (− 0.3, 1.2)	0.2
SDQ behavior	0.1 (− 0.7, 1.0)	0.7	0.2 (− 0.7, 1.1)	0.6
SDQ hyperactive	− 0.8 (− 2.1, 0.5)	0.2	**1.2 (0.2, 2.3)**	**0.02**
SDQ peers	0.1 (− 0.7, 1.0)	0.8	0.1 (− 0.8, 1.0)	0.8
SDQ prosocial	− 0.3 (− 1.4, 0.7)	0.5	− **0.9 (**− **1.7, **− **0.2)**	**0.02**
Screen-time (h/week)	1.3 (− 3.8, 6.3)	0.6	**2.9 (0.3, 5.6)**	**0.03**
Physical activity-score	0.6 (− 1.2, 2.3)	0.5	0.04 (− 1.8, 1.9)	0.96

P-values <0.05 are in bold and signify a statistically significant difference

Models were adjusted for age of child, maternal educational attainment and maternal nationality

KINDL-Scores: Higher values indicate more quality of life

SDQ-Scores: Higher values indicate more difficulties, except for SDQ-prosocial-score, where it is the inverse. Physical activity-score: Higher scores indicate more physical activity

Group 2 (Table 6): Interestingly, among boys in this group, five of the 15 assessed outcomes changed, though two of them were positive: physical activity increased by 1.2 (95% CI: 0.01, 2.4), and the SDQ subscale *emotion* showed less difficulties (b: − 0.8, 95% CI: − 1.2, − 0.3). However, screen time substantially increased, self-esteem dropped remarkably, and more hyperactive problems were found (Table 6). In contrast, among girls, screen time was reduced by 2 h per week (95% CI: − 3.8, − 0.3); however, there were several health-related outcomes among girls that were substantially negatively impacted by the time during vs. before the pandemic.

**Table 6:** Results of adjusted mixed models in Group 2: Differences in means comparing pre- and during pandemic outcomes of child health in children living in high housing density, n = 148 girls (n = 441 observations: n = 147 during pandemic), n = 131 boys (n = 454 observations: n = 169 during pandemic)

Outcomes of child health	Boys		Girls	
	Difference in means (95% CI)	p-value	Difference in means (95% CI)	p-value
KINDL total score	− 1.5 (− 5.2, 2.3)	0.4	− **4.5 (**− **8.3, **− **0.8)**	**0.03**
KINDL family	− 0.8 (− 5.8, 4.3)	0.8	− **5.2 (**− **10.2, **− **0.2)**	**0.04**
KINDL physical well-being	0.3 (− 5.7, 5.1)	0.9	2.3 (− 4.9, 9.5)	0.5
KINDL emotional well-being	1.8 (− 4–0, 7.6)	0.5	− **5.5 (**− **10.6, **− **0.3)**	**0.04**
KINDL self-esteem	− **6.3 (**− **12.2, **− **0.4)**	**0.03**	− 3.7 (− 9.5, 2.2)	0.2
KINDL friends	0.1 (− 6.5, 6.7)	0.96	− **8.5 (**− **15.4, **− **1.6)**	**0.02**
KINDL school	1.2 (− 3.9, 6.3)	0.7	− **7.3 (**− **13.8, **− **0.8)**	**0.035**
SDQ total score	0.4 (− 1.3, 2.1)	0.7	**2.2 (0.5, 4.0)**	**0.01**
SDQ emotion	− **0.8 (**− **1.2, **− **0.3)**	**0.004**	0.5 (− 0.1, 1.1)	0.057
SDQ behavior	0.1 (− 0.5, 0.7)	0.8	0.4 (− 0.2, 1.1)	0.2
SDQ hyperactive	**0.9 (0.08, 1.8)**	**0.03**	0.9 (− 0.03, 1.7)	0.08
SDQ peers	− 0.08 (− 0.8, 0.6)	0.8	0.3 (− 0.3, 0.9)	0.3
SDQ prosocial	0.3 (− 0.4, 1.0)	0.4	− 0.3 (− 1.1, 0.4)	0.2
Screen-time (h/week)	**4.0 (1.4, 6.6)**	**0.003**	− **2.0 (**− **3.8, **− **0.3)**	**0.02**
Physical activity-score	**1.2 (0.01, 2.4)**	**0.049**	1.0 (− 0.3, 2.3)	0.1

P-values <0.05 are in bold and signify a statistically significant difference

Models were adjusted for age of child, maternal educational attainment and maternal nationality

KINDL-Scores: Higher values indicate more quality of life

SDQ-Scores: Higher values indicate more difficulties, except for SDQ-prosocial-score, where it is the inverse. Physical activity-score: Higher scores indicate more physical activity

Group 3 (Table 7): Boys in this group showed a substantial hyperactive problem (SDQ *hyperactive* + 1.1 (95% CI: 0.4, 1.8), though none of the other outcomes were affected by the pandemic in boys. In girls, four outcomes of health-related quality of life (KINDL overall, subscales *family*, *friends*, and *school*) were negatively impacted, with a remarkable loss found in the subscale *friends* (− 12.5 (95% CI: − 17.8, − 6.9).

**Table 7:** Results of adjusted mixed models in Group 3: Differences in means comparing pre- and during pandemic outcomes of child health in children with mothers working ≥ 5 days/week, n = 150 girls (n = 414 observations: n = 145 duing pandemic), n = 115 boys (n = 367 observations: 131 during pandemic)

Outcomes of child health	Boys		Girls	
	Difference in means (95% CI)	p-value	Difference in means (95% CI)	p-value
KINDL total score	0.05 (− 3.7, 3.8)	0.98	− **5.8 (**− **9.1, **− **2.5)**	** < 0.001**
KINDL family	0.1 (− 4.8, 5.11)	0.96	− **5.5 (**− **10.2, **− **0.8)**	**0.02**
KINDL physical well-being	2.1 (− 3.4, 7.7)	0.4	− 2.4 (− 7.5, 2.7)	0.4
KINDL emotional well-being	− 1.0 (− 7.2, 5.1)	0.7	− 3.5 (− 8.0, 0.9)	0.1
KINDL self-esteem	− 1.5 (− 6.4, 3.4)	0.6	− 4.6 (− 10.6, 1.4)	0.1
KINDL friends	− 3.6 (− 10.7, 3.6)	0.3	− **12.5 (**− **17.8, **− **6.9)**	** < 0.001**
KINDL school	− 0.9 (− 6.6, 4.7)	0.7	− **6.2 (**− **10.8, **− **1.5)**	**0.01**
SDQ total score	1.1 (− 0.6, 2.7)	0.2	0.06 (− 1.2, 1.3)	0.9
SDQ emotion	− 0.4 (− 1.0, 0.2)	0.2	− 0.06 (− 0.6, 0.5)	0.8
SDQ behavior	0.02 (− 0.6, 0.6)	0.96	0.1 (− 0.4, 0.6)	0.6
SDQ hyperactive	**1.1 (0.4, 1.8)**	**0.004**	0.4 (− 0.3, 1.0)	0.3
SDQ peers	0.3 (− 0.3, 1.0)	0.96	− 0.2 (− 0.7, 0.23)	0.3
SDQ prosocial	0.6 (− 0.2, 1.3)	0.1	− 0.2 (− 0.8, 0.5)	0.6
Screen-time (h/week)	− 0.5 (− 3.1, 2.2)	0.7	0.03 (− 1.6, 1.6)	0.97
Physical activity-score	0.5 (− 0.9, 1.9)	0.5	0.8 (− 0.4, 2.0)	0.2

P-values <0.05 are in bold and signify a statistically significant difference

Models were adjusted for age of child, maternal educational attainment and maternal nationality

KINDL-Scores: Higher values indicate more quality of life

SDQ-Scores: Higher values indicate more difficulties, except for SDQ-prosocial-score, where it is the inverse. Physical activity-score: Higher scores indicate more physical activity

## Discussion

The results of this cohort study suggested that the health of children aged 6 to 9 years is possibly impacted by the COVID-19 pandemic, with adverse consequences differing by gender and very likely by the pre-pandemic family situation. In particular, girls from mothers with anxiety or depression symptoms may have suffered substantially during the COVID-19 pandemic. Boys showed fewer adverse trajectories, and it needs to be further assessed which factors exactly are behind the (socio-economic) factors, such as maternal working habits and limited living space, when analyzing the effect of the pandemic on children’s health.

The aim of our study was to contribute to a better understanding of the impact of the COVID-19 pandemic on mental health, quality of life, and lifestyle patterns of children under the consideration of the family and living situation of the child. These results help to identify groups at special risk for adverse trajectories and determine the different needs of children during a pandemic, as well as during the long phase out of the pandemic back to normality. Our results can help decision-makers develop quick and fit-for-purpose health-promotion strategies that target different family situations and vulnerable groups.

### Pre-pandemic family situation

It was shown that in already vulnerable children and those with financial strain, the COVID-19 pandemic aggravates mental health problems [11, 47]. This aligns with the theoretical framework from Prime, Wade, and Brown [29] and the results of a path analysis from Fosco et al. [48] Both show how a pandemic can disrupt family functioning and how pre-pandemic family conditions relate to coping-mechanisms during a pandemic. Given that the effect of financial strain on children’s mental health may be mediated by parent’s mental health [11] and the likelihood that domestic violence and abuse will increase during public health emergencies [49], there is a need to disentangle the complex associations between the impact of a pandemic, parent’s mental health, and children’s mental health. That being said and given the fact that a pandemic has harmful effects in adults as well [1, 50], we developed Directed Acyclic Graphs depicting the possible associations between variables (Additional file 2: Figs. S1–S3).

### Maternal health

Boys whose mothers scored high on HADS-D or HADS-A (Group 1) showed a strong loss of health-related quality of life in the subscale *friends*. Among girls in Group 1, many outcomes assessed changed negatively, and it seems that girls in this group suffered substantially more under the pandemic than boys. The domains *family*, *emotional well-being* and *physical well-being* were especially impacted, and the increased screen-time in girls was remarkable. It is well known that maternal mental health conditions can have consequences on child mental health [41, 51, 52]. It was further shown that adults with pre-pandemic mental health conditions have a greater risk for severe mental health issues during the pandemic [53–56]. For example Thompson et al. [47] found that (1) pre-pandemic familial contextual risk, and Fosco et al. [48] that (2) pre-pandemic emotional distress are associated with child internalizing and externalizing problems during the pandemic. Also Richard et al. revealed that having parents with average to poor mood compared to good mood was associated with being severely impacted by the pandemic [57]. Further, Gruhn et al. showed that care-givers' depression symptoms, as well as family conflict predicted levels of child depression symptoms during the pandemic [58]. Those results align with the conceptual framework underlying this work depicting that caregivers mental health influences child adjustment [29]. However, Khoury, Kaur, and Gonzalez found in a cross-sectional analysis—potentially prone to reverse causation—that parental support and parental mental health or distress was not associated with child internalizing or externalizing problems [59]. It was further shown that family routines were not associated with child mental health during the pandemic [48]. However, authors assume that this particular finding might relate the used measure for family routine, which focused on patterns of day-to-day family interactions, such as regular family meals, bedtime routines, and family activities [48]. In the light of day-care-closures, home schooling, and loss of daily activities those routines were possible less important for child health in the early phase of the pandemic [48]. Since there is further evidence that higher levels of pre-pandemic parental stress, anxiety or depression is associated with child mental health [60], we conclude that this group marks the group on which researchers, social workers, and public health policy makers should probably focus most to minimize the detrimental effects of crises such as a pandemic.

### Living space

It has been shown that limited living space during the pandemic (< 20m^2^ per person^26^) negatively affected quality of life, mental health, and anxiety among children, [2, 27, 33], youth [34], and adults [35, 60]. One reason could be that during a lockdown, spare housing density might be even more difficult to handle than under “normal conditions” [61]. It can be further explained by the housing-health relationship [30–32]. Our results strengthened those findings: **Group 2** comprised all children living in limited living space (i.e., high housing density for at least two of five consecutive years). In this group, boys showed the largest increase in screen-time, whereas girls showed a substantial reduction of approximately 2 h per week. It has to be further assessed why the results on screen-time are contrary regarding gender. Boys also showed a remarkable loss in self-esteem, which has been shown to be associated with screen-time, however, in slightly older children (13 years) [62]. Yet, among boys, physical activity improved, and fewer emotional problems were found. It can be hypothesized that these components are associated with each other especially during a pandemic [63]. Among girls, the increase in the SDQ total score strongly indicates substantial mental health problems comparing the time during vs. before the pandemic. Those results are in line with the often reported gender differences regarding the pandemic responses [21, 64–66], especially decreased mental health among girls. [26, 66]

### Maternal working habits

In our analysis, there was one group defined by the working habits of the mother: **Group 3** included all children whose mother worked five or more days per week for at least one year. Based on the theoretical framework from Prime, Wade, and Brown [29] we assumed that in those families the pandemic led to drastic changes in family functioning as the daily routines of mothers and children enormously changed. High adjustment of children across several domains, such as communication, and organization of the whole family processes was needed. [29] Additional file 2: Figure S2 also depicts the applied underlying framework. Boys in this group showed a substantial increase in hyperactive problems and girls showed a remarkable loss of health-related quality of life. Both findings are plausible when considering the child’s necessary adaption noted above. It needs however, further research whether such adaption can lead to hyperactive problems [67, 68] and reduced quality of life. Yet, having a poor parent–child relationship during the pandemic is associated with being more at risk for adverse outcomes [57]. Hyperactive problems are more common in boys than in girls [69] and should possibly be considered when evaluating the causal pathways between a pandemic and a child’s pandemic response/mental health. Both findings strongly indicate the need for further research also shedding light on this specific pre-pandemic family situation. It might be possible that maternal working habits moderate the detrimental effects of a pandemic on a child’s health.

## Limitations

The interpretation of our results is limited by sample size and the resulting lack of power. In addition, we had a high proportion of families with high educational attainment of the mother at study entry, which is representative of the local population, but in families with low education and migration background, loss to follow-up was higher, especially during the first year of follow-up.

When interpreting our results, it must be taken into account that assessing the impact of the COVID-19 pandemic on child health was not an a-priori hypothesis of Ulm SPATZ Health. We only had the routinely collected study data and no special pandemic-related questionnaires. On the other hand, using routinely assessed data could be a strength of the longitudinal SPATZ study, as it is not an intended COVID-19 pandemic-related study, hence preventing several forms of bias arising from selection and awareness in participants. Meaning, especially for possible pandemic effects, selection bias, recall bias, and conscious bias, can be minimized when routinely assessed data are used.

Since every group was analyzed separately, it was not the goal of the analysis to perform intergroup comparisons, for which a different statistical analysis would have been necessary, and therefore differences between different groups should be interpreted carefully. Furthermore, the analysis does not allow us to draw causal conclusions or to identify independent predictors for adverse trajectories related to a specific family situation.

## Conclusion

Despite the above mentioned limitations, we conclude that the health (and behavior) of primary school children may be impacted by the COVID-19 pandemic, with adverse consequences possibly differing by gender and very likely by pre-pandemic family situations. Especially in girls having a mother with depression or anxiety symptoms, the adverse consequences of the pandemic on mental health seem to be aggregated.

## Supplementary Information

**Additional file 1: Table S1.** Maternal data for further follow-up waves (T10, and T11 during pandemic). **Table S2.** Children data for waves (T5, T6, T8, and T10).

**Additional file 2: Figure S1.** Directed Acyclic Graph depicting how maternal pre-pandemic mental health is possibly associated with child’s mental health during a pandemic. **Figure S2.** Directed Acyclic Graph depicting how pre-pandemic high housing density (as a social determinant of health) is possibly associated with child’s mental health during a pandemic. **Figure S3.** Directed Acyclic Graph (DAG) depicting how maternal pre-pandemic working habits are possibly associated with child’s mental health during a pandemic. This DAG is explorative, as we assumed that the pandemic related restrictions/changes might cause a bigger change in daily routines of those mothers who were used to go to work on a daily basis (every day).

## Data Availability

The datasets generated during and/or analyzed during the current study are not publicly available due to ethical restrictions regarding data protection issues and the study-specific consent text and procedure, but anonymized data are available from the corresponding author upon reasonable request. Supplemental results are available upon request.

## Abbreviations

SPATZ: Ulm SPATZ Health Study
OR: Odds ratio
CI: Confidence interval
SD: Standard deviation
SDQ: Strengths and Difficulties Questionnaire
vs.: Versus
SES: Socio-economic status
h: Hours
FU: Follow-up
DAG: Directed Acyclic Graph
