# Supplementary material for: Trajectories of child mental health, physical activity and screen-time during the COVID-19 pandemic considering different family situations: results from a longitudinal birth cohort
Source: Child Adolesc Psychiatry Ment Health. 2023 Mar 10;17:36. doi: 10.1186/s13034-023-00581-3 (PMC9999332; doi:10.1186/s13034-023-00581-3)
Supplement: Supplementary file 1 — Additional file 1: Table S1. Maternal data for further follow-up waves (T10, and T11 during pandemic). Table S2. Children data for waves (T5, T6, T8, and T10). [file 13034_2023_581_MOESM1_ESM.docx]

**Additional file 1: Table S1**. Maternal data for further follow-up waves (T10, and T11 during pandemic).

|  | | **T10** | **T11** |
| --- | --- | --- | --- |
| Duration of data sampling for specific follow-up (FU) wave | | 04/20 - 07/21 | 04/21 - 07/22 |
| **Mothers, N** | | **321** | **244** |
|  |  |  |  |
| **Age** (n observations) | | (321) | (244) |
|  | Mean (SD) | 41.4 (4.4) | 42.4 (4.6) |
| **Number of regular weekly working days, N (%)** | | (321) | (244) |
|  | <5 days/week | 182 (56.7) | 144 (59.0) |
|  | ≥5 days/week | 125 (39.3) | 91 (37.3) |
|  | Missing | 13 (4.1) | 9 (3.7) |
| **Housing density, m^2^/person** | | - | - |
|  | Mean (SD), Median (Q1, Q3) | - | - |
| **Hospital Anxiety and Depression Scale (HADS)** | | - | (239) |
|  | Mean (SD),  Median (Q1, Q3) | - | 10.5 (6.5),  9.0 (6.0, 15.0) |
|  | HADS_A ≥8, N (%) | - | 85 (35.6) |
|  | HADS_D ≥8, N (%) | - | 36 (15.1) |

**Additional file 1: Table S2.** Children data for waves (T5, T6, T8, and T10).

|  | | **T5** (3 years) | **T6** (4 years) | **T8** (6 years) | **T10** (8 years) |
| --- | --- | --- | --- | --- | --- |
| Duration of data sampling for specific follow-up (FU) wave | | 04/15 – 07/16 | 04/16 - 07/17 | 04/18 - 07/19 | 04/20 - 07/21 |
| **Children, N** | | **649** | **567** | **422** | **328** |
| Set of twins | | 24 | 21 | 14 | 7 |
| **Age** (n observations) | | (649) | (567) | (422) | (328) |
|  | Mean (SD) | 3.3 (0.8) | 4.1 (0.1) | 6.1 (0.1) | 8.1 (0.1) |
| **Health related quality of life (KINDL)^a^** | | - | (525) | (365) | (312) |
| **Total score,** Mean (SD),  Median (Q1, Q3) | | - | 80.5 (0.3),  81.3 (76.0, 86.5) | 80.3 (0.4),  80.2 (76.0, 85.4) | 82.1 (0.6),  83.3 (77.1, 88.5) |
| N |  | - | (563) | (370) | (327) |
|  | Family, Mean (SD),  Median (Q1, Q3) | - | 82.2 (0.5),  81.3 (75.0, 87.5) | 81.8 (0.6),  81.3 (75.0, 7.5) | 81.4 (0.7),  81.3 (75.0, 93.8) |
| N |  | - | (560) | (371) | (325) |
|  | Physical well-being, Mean (SD), Median (Q1, Q3) | - | 83.2 (0.6),  87.5 (75.0, 93.8) | 84.1 (0.7),  87.5 (75.0, 93.8) | 87.2 (0.7),  87.5 (81.3, 100.0) |
| N |  | - | (564) | (370) | (327) |
|  | Emotional well-being,  Mean (SD), Median (Q1, Q3) | - | 87.5 (0.5),  87.5 (81.3, 93.8) | 85.3 (0.6),  87.5 (81.3, 93.8) | 83.3 (0.7),  87.5 (75.0, 93.8) |
| N |  | - | (554) | (370) | (326) |
|  | Self-esteem, Mean (SD),  Median (Q1, Q3) | - | 79.9 (0.5),  81.3 (75.0,87.5) | 79.8 (0.7),  78.1 (75.0, 87.5) | 77.6 (0.9),  75.0 (68.8, 87.5) |
| N |  | - | (557) | (369) | (321) |
|  | Friends, Mean (SD),  Median (Q1, Q3) | - | 81.4 (0.5),  81.3 (75.0, 87.5) | 81.5 (0.7),  81.3 (75, 87.5) | 78.6 (0.9),  81.3 (75.0, 87.5) |
| N |  | - | (540) | (366) | (318) |
|  | School, Mean (SD),  Median (Q1, Q3) | - | 68.7 (0.3),  68.8 (62.5, 75.0) | 69.3 (0.4),  68.8 (62.5, 75.0) | 84.0 (0.8),  87.5 (75.0, 93.8) |
| **SDQ Strengths and Difficulties Questionnaire^b^** | |  | (557) | (418) | (326) |
| **Total Difficulties Score**, Mean (SD), Median (Q1, Q3) | | - | 7.6 (0.2),  7.0 (4.0, 10.0) | 6.7 (0.2),  6.0 (3.0, 10.0) | 7.3 (0.3),  6.0 (3.0,10.0) |
|  | SDQ emotion, Mean (SD) | - | 1.3 (0.06) | 1.4 (0.07) | 1.5 (0.1) |
|  | SDQ behavior, Mean (SD) | - | 2.1 (0.07) | 1.8 (0.08) | 1.6 (0.09) |
|  | SDQ hyperactive, Mean (SD) | - | 2.9 (0.09) | 2.6 (0.1) | 3.1 (0.1) |
|  | SDQ peers, Mean (SD) | - | 1.3 (0.07) | 1.0 (0.07) | 1.0 (0.08) |
|  | SDQ prosocial, Mean (SD) | - | 7.7 (0.07) | 8.1 (0.08) | 8.1 (1.0) |
| **Physical Activity Score^c^** | | - | (565) | (420) | (327) |
|  | Mean (SD),  Median (Q1, Q3) | - | 0.06 (0.1),  -1.0 (-1.0, 3.0) | 1.3 (0.1),  1.0 (-1.0, 3.0) | 1.1 (0.2),  1.0 (-1.0, 3.0) |
| **Screen-time (h/week)^d^** | | - | (560) | (415) | (327) |
|  | Mean (SD),  Median (Q1, Q3) | - | 5.2 (0.2),  3.5 (1.4, 7.0) | 6.4 (0.3),  4.5 (2.4, 8.4) | 9.3 (0.4),  7.5 (4.5, 13.0) |
| **Time spent with books (h/week)^e^** | | - | (560) | (415) | (327) |
|  | Mean (SD)  Median (Q1, Q3) | - | 7.5 (0.2),  7.0 (3.9, 9.5) | 6.7 (0.2),  6.0 (3.5, 8.5) | 6.3 (0.2),  4.9 (3.5, 8.0) |

h=hours; SD=standard deviation. Q1=First Quartil; Q3=Third Quartil.

^a^ KINDL questionnaire: higher values indicate more health-related quality of life.

^b^ Strengths and Difficulties Questionnaire (SDQ): higher values indicate more emotional and behavioral difficulties, except for SDQ-prosocial-score, where it is the inverse.

^c^ Physical activity-score: Higher score more physical active. The score indicates how many items answered with "physically active" outweigh the items answered with "physically inactive". Score from -7 to +7.

^d^ Including time spent with TV/DVD (also via computer/smartphone), time spent with computer games/game consoles (also via smartphone), time spent with other use of internet/computer (also via smartphone).

^e^ Either read by themselves or read to them by someone else.
