# Supplementary material for: Trajectories of child mental health, physical activity and screen-time during the COVID-19 pandemic considering different family situations: results from a longitudinal birth cohort
Source: Child Adolesc Psychiatry Ment Health. 2023 Mar 10;17:36. doi: 10.1186/s13034-023-00581-3 (PMC9999332; doi:10.1186/s13034-023-00581-3)
Supplement: Supplementary file 2 — Additional file 2: Figure S1. Directed Acyclic Graph depicting how maternal pre-pandemic mental health is possibly associated with child’s mental health during a pandemic. Figure S2. Directed Acyclic Graph depicting how pre-pandemic high housing density (as a social determinant of health) is possibly associated with child’s mental health during a pandemic. Figure S3. Directed Acyclic Graph (DAG) depicting how maternal pre-pandemic working habits are possibly associated with child’s mental health during a pandemic. This DAG is explorative, as we assumed that the pandemic related restrictions/changes might cause a bigger change in daily routines of those mothers who were used to go to work on a daily basis (every day). [file 13034_2023_581_MOESM2_ESM.docx]

**Maternal pre-pandemic mental health**

**
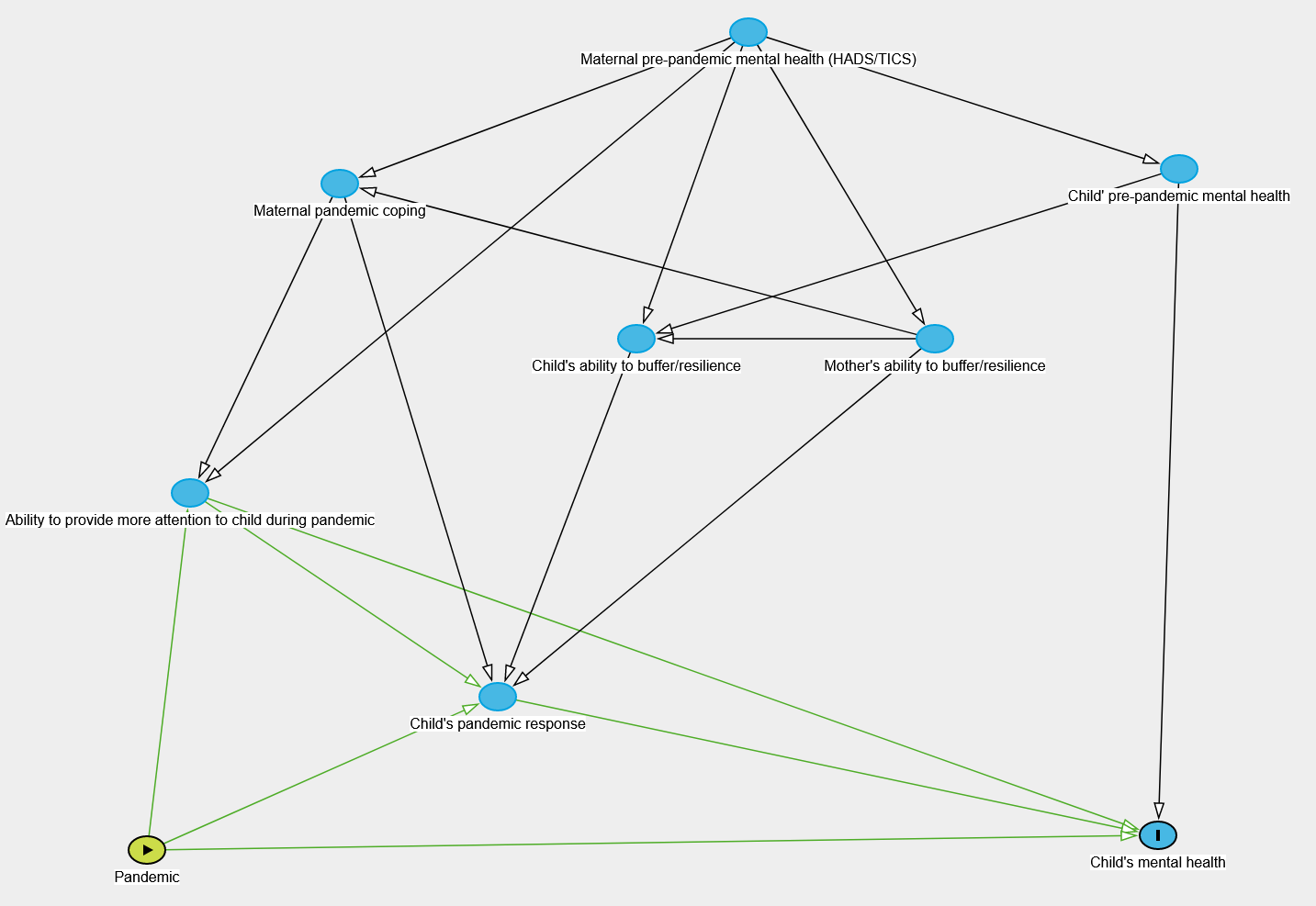
Additional file 2: Figure S1.** Directed Acyclic Graph depicting how maternal pre-pandemic mental health is possibly associated with child’s mental health during a pandemic.

**Housing density as a social determinant of health**

**
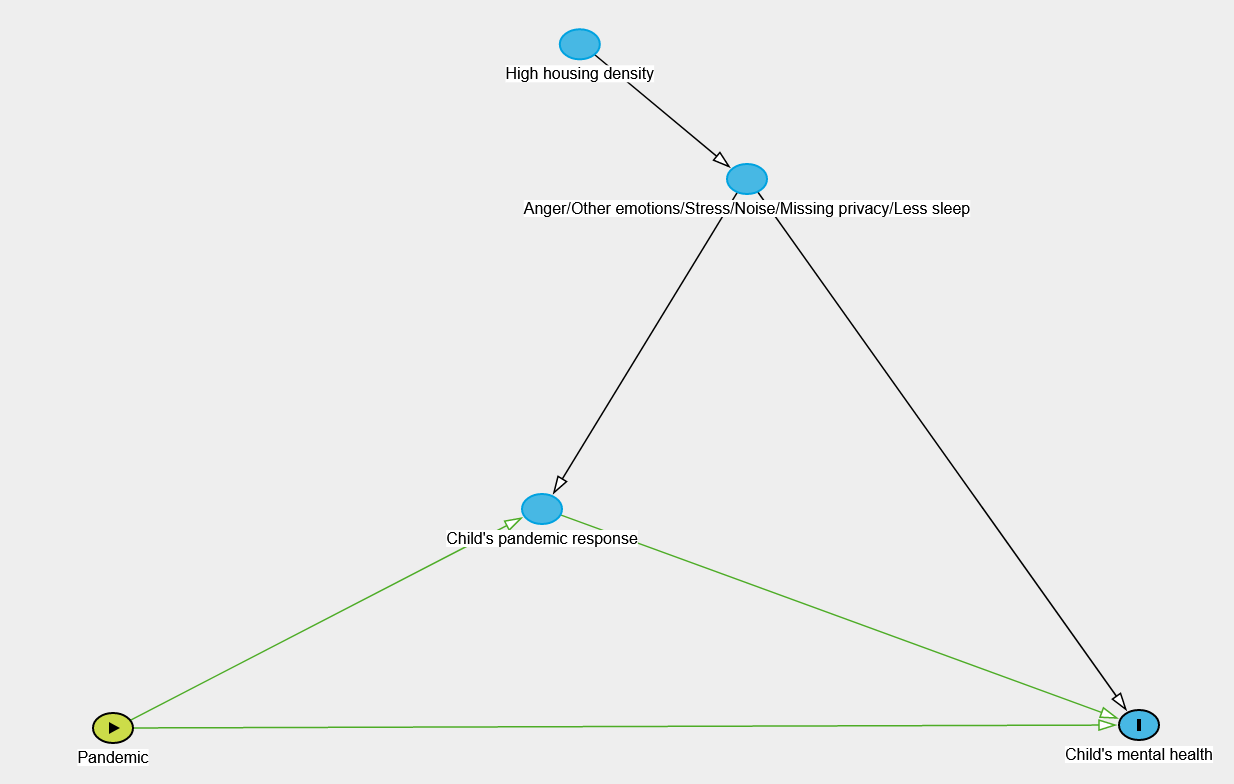
**

**Additional file 2: Figure S2.** Directed Acyclic Graph depicting how pre-pandemic high housing density (as a social determinant of health) is possibly associated with child’s mental health during a pandemic.

**Maternal working days**
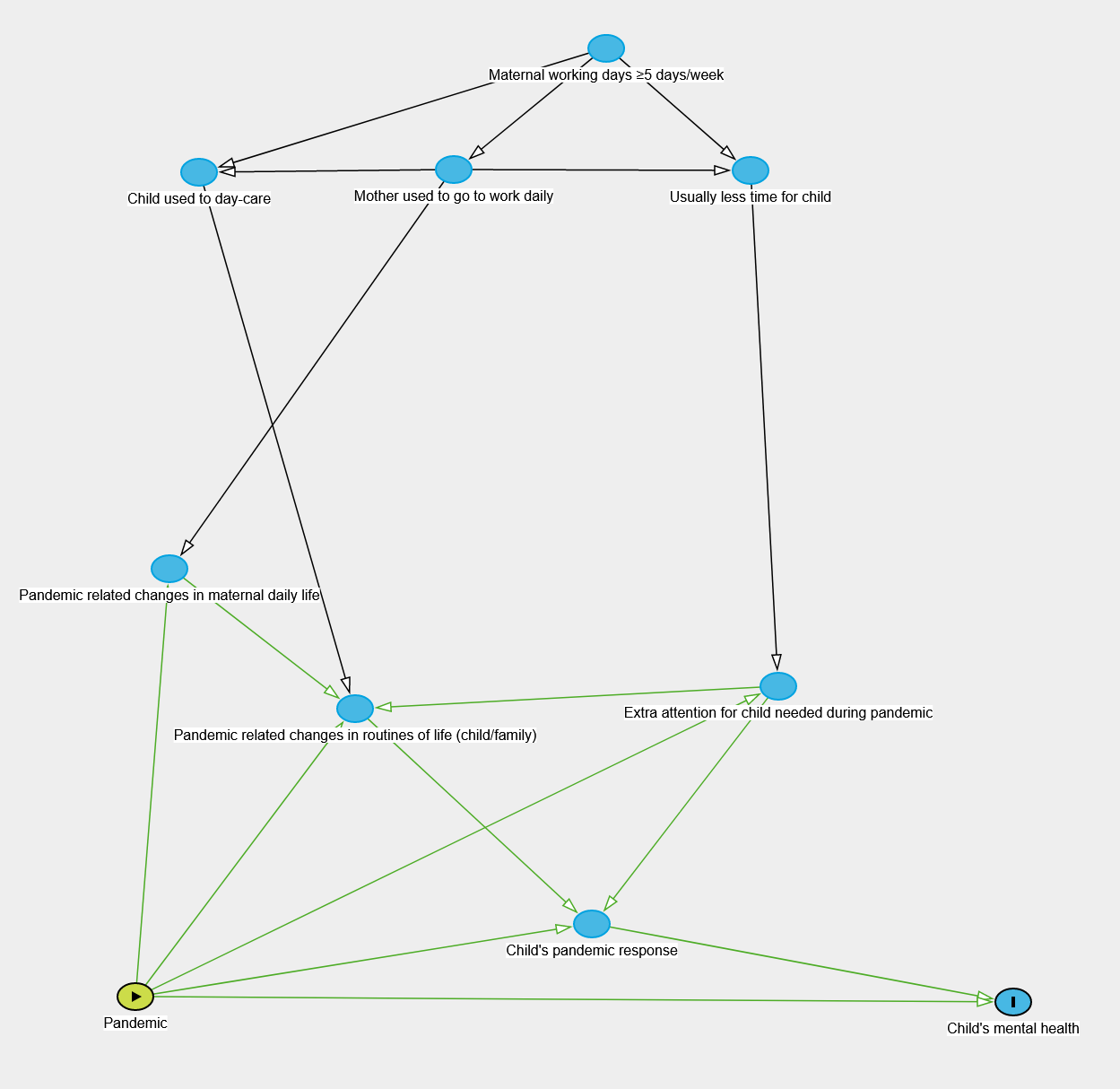


**Additional file 2: Figure S3**. Directed Acyclic Graph (DAG) depicting how maternal pre-pandemic working habits are possibly associated with child’s mental health during a pandemic. *This DAG is explorative, as we assumed that the pandemic related restrictions/changes might cause a bigger change in daily routines of those mothers who were used to go to work on a daily basis (every day).*
